# Assess the diversity of gut microbiota among healthy adults for forensic application

**DOI:** 10.1186/s12934-022-01769-6

**Published:** 2022-03-24

**Authors:** Shuangshuang Wang, Feng Song, Haoyu Gu, Zhilong Shu, Xiaowen Wei, Ke Zhang, Yuxiang Zhou, Lanrui Jiang, Zefei Wang, Jienan Li, Haibo Luo, Weibo Liang

**Affiliations:** 1 Department of Forensic Genetics, West China School of Basic Medical Sciences & Forensic Medicine, Sichuan University, Chengdu, Sichuan China; 2 Department of Forensic Science, School of Basic Medical Sciences, Central South University, Changsha, Hunan China

**Keywords:** Gut microbiota, Forensic science, Microbiota diversity, 16S rRNA

## Abstract

**Background:**

Human gut microbiota is individually unique that hints the microbiota in fecal traces left in the crime scene could act as a potential biomarker for forensic personal identification. Next-generation DNA sequencing and bioinformatic analysis of fecal samples are revolutionizing our insights into gut microbial communities. While the formation of the gut microbiota is known to be multifactorial, it is unclear whether these characteristics can be applied to forensic applications. Therefore, the gut microbiota of healthy adults with different traits was investigated in this study.

**Results:**

Based on the STAMP analysis of each study group, the difference in gut microbiota composition of male and female subjects was observed. The male group was characterized by taxa in the phylum *Proteobacteria*, while the female group was described by *Synergistetes* phylum. The gut bacterial community assembly mechanism was mainly affected by the deterministic process. In addition, gut microbiota composition showed meaningful discrimination in each of the BMI groups. At the phylum level, in male subjects, increased representative phyla were *Patescibacteria* (p < 0.05) in the underweight group and *Bacteroidetes* (p < 0.05) in the normal-weight group, while in the female group, the significantly different phyla were *Bacteroidetes*, *Firmicutes,* and *Actinobacteria*. At the genus level, 44 unique genera were found to be significantly distinct across BMI study groups. By Fisher’s Linear Discriminant Analysis, ninety-four point four percent (94.4%) of original BMI grouped subjects were correctly classified. The linear regression analysis model showed an accuracy of seventy-four percent (74%) in predicting body type.

**Conclusion:**

In conclusion, subjects with different individual characters have specific gut microbiota, and can be discriminated by bioinformatics methods, suggesting it is promising to apply gut microbiota to forensic personal identification.

**Supplementary Information:**

The online version contains supplementary material available at 10.1186/s12934-022-01769-6.

## Background

Identifying the personal characteristics of the trace material left at the crime scene is an important task of forensic science. Feces, as trace evidence of human activity, can be abandoned at the offense scene [1] and is not susceptible to incompletely removed outdoors. The feces sample contains host-microbial profiles that could provide insight into valuable individual information on digestion or diet habits as investigative clues and trial basis. In recent years, with the development of next-generation DNA sequencing technology and bioinformatic methods, the application of human microbiota to forensic personal identification has been studied in the saliva microbiome [2, 3], skin microbiome [4–7], hair microbiome [8], etc. However, the studies about fecal material have focused on cell type identification to determine the cellular origin of samples/DNA profiles for forensic practice [9, 10], and there is little attention on gut microbiota for forensic personal identification. Recent studies reported the gut microbiota of subjects may be specific [11, 12] that declared remarkable personal characters of gut microbiota across individuals. Despite it is known that human gut microbiota is individually unique, it is unclear whether gut microbiota can discriminate different subjects or predict personal signature. With increased concern on forensic potential of using the human microbiome, it is essential to investigate the gut microbiota of subjects with different traits.

This study was an attempt to explore the potential of individualized gut microbiota for forensic personal identification. The gut microbiota is acquired by the environment from birth and can act as a genetically determined property, shaped by and interacted with the host [13–15], and the gut microbiome is regarded to be shaped by human genetics [16]. A series of other factors also affect the composition of the human microbiota, including external factors and internal factors, like food, pathogens, drugs, and endocrine ingredients [17]. Since the highly personalized gut microbiota is multifactorial, it is important to seek the elements associated with the composition of gut microbiota for forensic application. Recent studies have revealed a sex bias across microbiome-associated diseases, while another report has also shown sex differences in the gut microbiota of the Japanese population [17, 18]. In addition, the gut microbiota is an important role in human physiology, making it be a significant factor in the development of obesity. A study of gut microbiota profiles of different weight individuals reveals distinctions of bacterial communities in Italian adults [19]. Therefore, this study targeted to the gut microbiota of Chinese adults, and the subjects were sub-grouped based on the individual traits to: (1) observe the differences of gut microbiota community composition in separate study groups, (2) use the variations of the gut microbiota composition to discriminate experimental subjects and to predict some personal signatures, and (3) explore the potential of individualized gut microbiota for using in forensic personal identification.

## Material and methods

### Sample collection and DNA extraction

54 unrelated participants, aged 20–30, who self-claimed that they had no intestinal diseases and had not used antibiotics in the past three months were enrolled. All subjects were local students from the same school and had lived in Chengdu for at least six months. After obtaining the informed consent, a total of 54 fecal samples and data which involves age, body weight, body height, and habitation were collected. The subjects contained 32 females and 22 males in our study. The Body Mass Index (BMI) of participants was calculated to divided the subjects into different obesity groups. BMI is calculated as weight (kg) divided by height squared (m^2^), expressed in kg/m^2^. The fecal collection was performed strictly under the following requirements: (1) avoid miscellaneous bacteria pollution in urine and toilet; and (2) use 5 ml sterilized disposable stool sample collection tubes to collect the middle section of the feces isolated from the air and then promptly stored at − 80 °C pending DNA extraction. Total Genomic DNA was isolated using the OMEGA Soil DNA Kit (M5635-02) (Omega Bio-Tek, Norcross, GA, USA), following the manufacturer’s instructions. Extracted DNA was quantified using a NanoDrop 2000 spectrophotometer (Thermo Fisher Scientific, Waltham, MA, USA). DNA was stored at − 20 °C prior to further analysis.

### PCR amplification and sequencing

The amplification targeted at the V3-V4 region of the 16S rRNA gene was performed with the forward primer 338F (ACTCCTACGGGAGGCAGCA) and the reverse primer 806R (GGACTACHVGGGTWTCTAAT). Sample-specific 7-bp barcodes were incorporated into the primers for multiplex sequencing. The components of PCR (25 μL) reaction were as follows: 5 μL of reaction buffer (5×), 5 μL of GC buffer (5×), 2 μL(2.5 mM) of dNTPs, 1 μL(10uM) of each forward primer and reverse primer, 2 μL of DNA Template, 8.75 μL of ddH_2_O, and 0.25 μL of Q5 DNA Polymerase. The thermal cycling conditions for the PCR amplification consisted an initial denaturation at 98 °C for 2 min, followed by 30 cycles of denaturation at 98 °C for 15 s, annealing at 55 °C for 30 s, extension at 72 °C for 30 s, with a final extension at 72 °C for 5 min. To remove any remaining contaminants and PCR artifacts, the purification of amplicons products was performed with Vazyme VAHTSTM DNA Clean Beads (Vazyme, Nanjing, China) according to the manufacture’s recommendations. The quality and quantity of amplicons were confirmed by 1.2% agarose gel electrophoresis and Quant-iT PicoGreen dsDNA Assay Kit (Invitrogen, Carlsbad, CA, USA). Purified amplicons were pooled in equal amounts, and pair-end 2 × 250 bp sequencing was performed using the Illumina NovaSeq platform with NovaSeq 6000 SP Reagent Kit (500 cycles) at Shanghai Personal Biotechnology Co., Ltd (Shanghai, China).

### Sequence, bioinformatics analysis, and visualization

The Illumina Novaseq platform is used for paired-end sequencing of community DNA fragments. Raw sequencing data was performed DADA2 [20] sequence denoising with QIIME2 [21] to demultiplex, quality filter, denoise, splice, merge and chimera remove according to the official tutorials (https://docs.qiime2.org/2019.4/tutorials/). Taxonomy was assigned to ASVs using the classify-sklearn naïve Bayes taxonomy classifier in the feature-classifier plugin [22] against the SILVA Release 132 Database (https://www.arb-silva.de/). The metrics of α diversity and β diversity were estimated using the diversity plugin. The α-diversity (microbial diversity within a sample): Chao1, observed_Species, Shannon were plotted as violin boxplot with Wlicox test using the R script. The β-diversity (microbial diversity between samples) was assessed using Bray–Curtis distances and visualized via principal coordinate analysis (PCoA). The comparison of gut bacterial community composition in each study group was analyzed with STAMP [23] software. The neural community model (NCM) was constructed with an R script [24]. The normalized stochasticity ratio (NST) was calculated to evaluate the relative importance of stochastic and deterministic processes in gut bacterial community assembly [25]. NST reflects the contribution of stochastic assembly relative to deterministic assembly, based on the magnitude of the difference between observed and null expectations, as a quantitative measure of stochasticity. The relative importance of deterministic and stochastic processes in different community constructions was quantified by comparing the numerical distribution of NST between sample pairs within different groups: the range of values of NST (0–1). If the NST of a group of communities is mainly distributed above 50% then the stochastic process is considered to dominate within that group of communities, conversely, if the NST value is below 50%, the deterministic process is considered to be dominant in that group of communities [25, 26]. NCM, a neutral-based process model, has been successfully applied to a wide range of ecological phenomena due to the effective ability to infer the stochastic processes acting on community assembly mechanisms [27, 28]. The Fisher’s Linear Discriminant Analysis and correlation analysis were performed in R. It warned six bacterial genera variables appeared to be constant within groups during the running process, so, the six bacteria were excluded during the Fisher’s Linear Discriminant Analysis. The ridge regression analysis was performed on SPSSPRO website (https://www.spsspro.com). To make the model concise and reduce noise in the dataset, the lasso regression was performed to remove the bacteria genera with a value of zero, and the remaining bacterial genera were used for linear regression. All processes of regression model construction were performed on RStudio with an R script.

## Results

### The overview of sequencing results and study group

5,726,427 raw reads were obtained from 54 fecal samples. After removing low-quality sequence reads, chimeras, and singletons, 3,265,754 clean reads were obtained. The length of clean reads ranged from 403 to 431 bp for each sample, with the average length of clean reads being 417 bp. The subjects were sub-grouped into male/female group according to the sex, and divided into three BMI groups according to the BMI value with the Chinese reference standard: underweight (L): BMI < 18.5 kg/m^2^, normal weight (N):18.5 kg/m^2^ ≤ BMI < 24.0 kg/m^2^, overweight (H): BMI ≥ 24.0 kg/m^2^.

### The sex-related gut microbiome

**Fig. 1:**
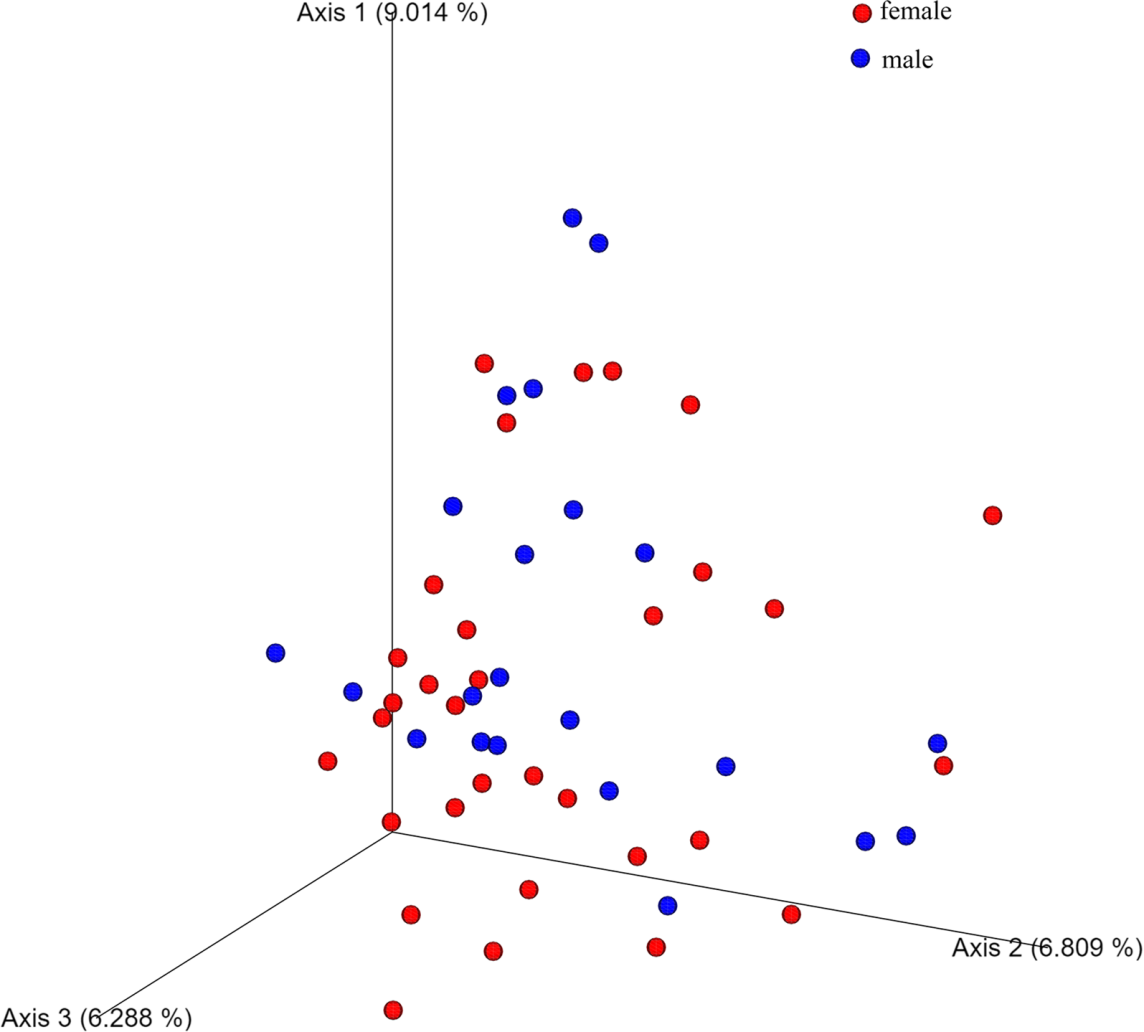
Principal coordinate analysis (PCoA) plots based on Bray–Curtis distance showing the gut microbiota compositions. The overall structure of gut bacterial community was different between male and female groups

**Fig. 2:**
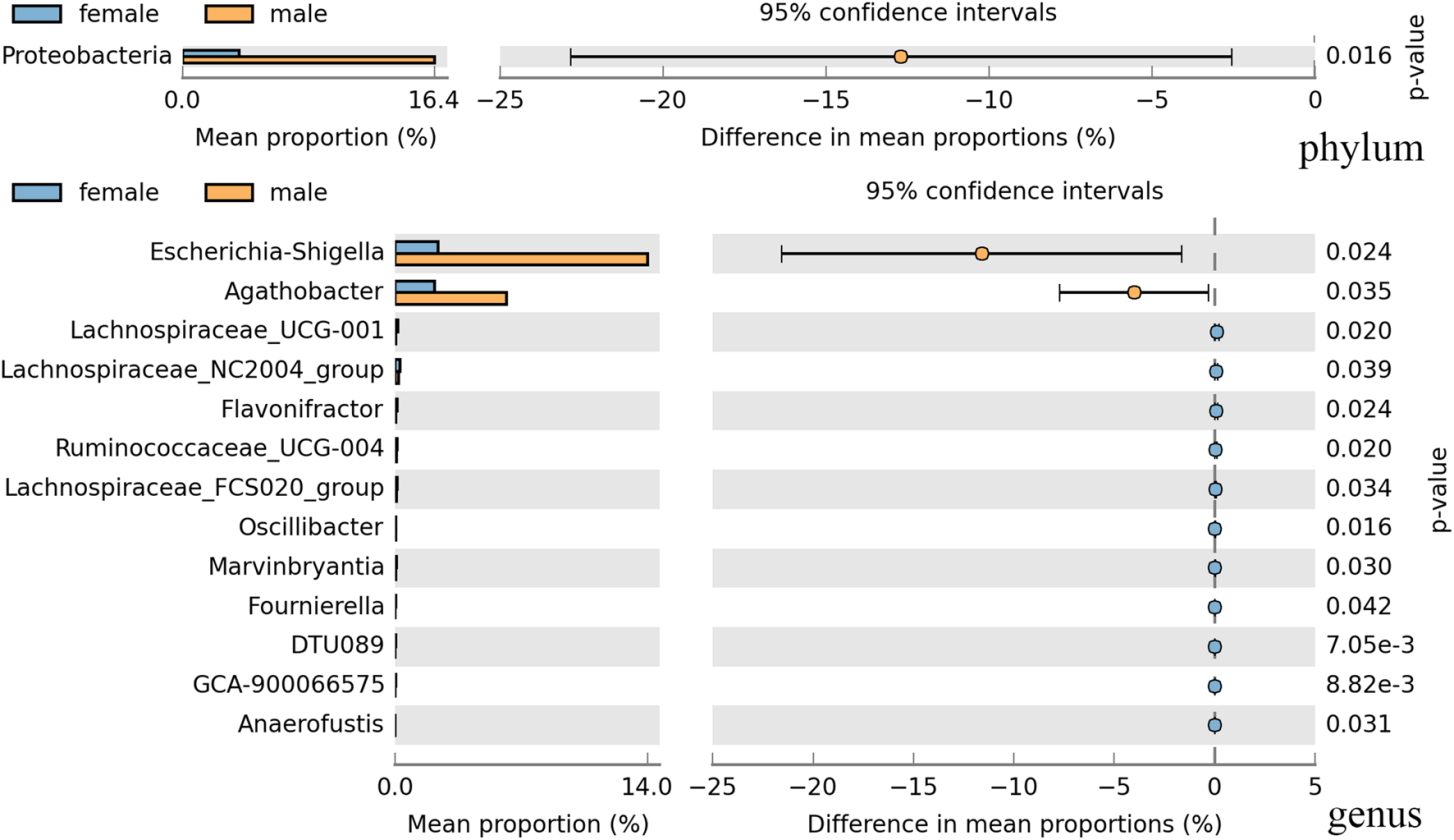
Gut microbiota comparison of male and female subjects at phylum and genus level. The phyla and genera with significant richness difference (*p* < 0.05, computed by STAMP) between the two groups were shown. *Proteobacteria* phylum showed a remarkable high abundance in male group, and 13 genera revealed difference in sex group

**Fig. 3:**
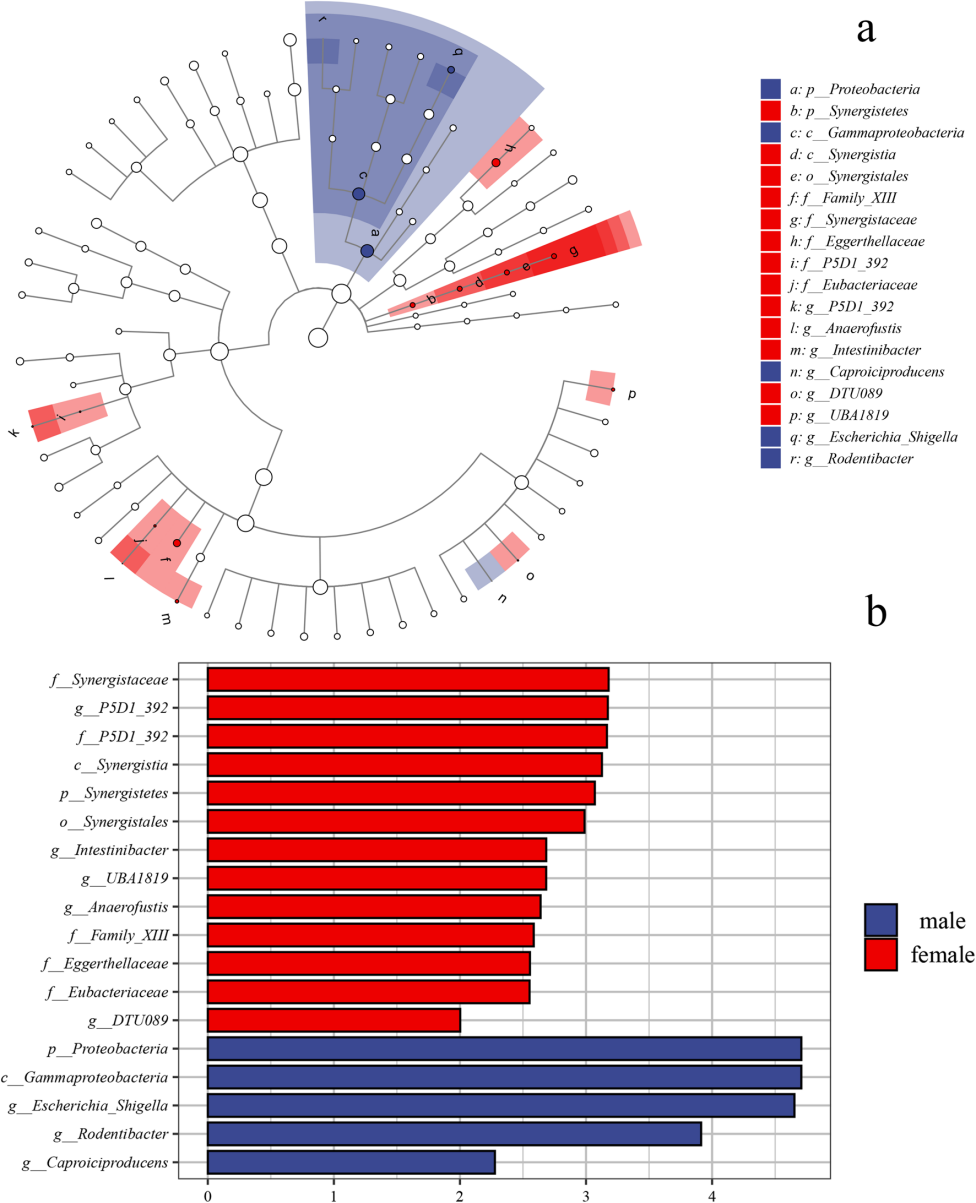
The results of Linear discriminant analysis effect size (LEfSe). **a** The cladogram of taxa showed significant difference between male and female gut microbiota. **b** The bar graph of LDA scores showed the taxa with statistics difference between two groups. The LDA threshold was 4. The male group was characterized by taxa in the phylum *proteobacteria*, while female group was characterized by taxa in the phylum *Synergistetes*

Initially, the overall gut microbial richness of male and female subjects was reflected by α diversity indices. The two sex groups did differ significantly in Chao1 index, the number of observed species (richness estimation) and Shannon index (evenness estimation) (Wlicox test, p = 0.029, p = 0.028, and p = 0.036, respectively), which were shown in Additional file 1: Fig. S1. Subsequently, the β diversity was assessed using Bray–Curtis distance and visualized by the principal coordinate analysis (PCoA). It showed the overall structure of the gut bacterial community was different between the male and female groups (Fig. 1). Then the difference in bacterial community composition was analyzed. At the phylum level, the predominant phyla were *Firmicutes, Bacteroidetes, Actinobacteria,* and *Proteobacteria* in the gut bacterial community (Additional file 1: Fig. S2). It was observed only *Proteobacteria* had remarkable differences between the two sex groups (Fig. 2). The difference of taxon in the gut bacterial community was also assessed at the genus level. In total, the genera with relatively high abundance were *Faecalibacterium, Bacteroides, Subdoligranulum, Escherichia-Shigella, Blautia, Prevotella_9, Agathobacter, Bifidobacterium, Roseburia,* and *Dialister* (Additional file 1: Fig. S2). The change in the gut bacterial community between the male and female group was manifested in Fig. 2, which showed a significant difference of 2 genera in male subjects and 11 genera in female subjects. The Linear discriminant analysis effect size (LEfSe) result showed 13 nested taxa in the female group and 5 in the male group, which were regarded to explain the difference between the two groups (Fig. 3). The male group was characterized by *proteobacteria* phylum, while the female group was characterized by *Synergistetes* phylum.

### Ecological assembly of bacterial community

**Fig. 4:**
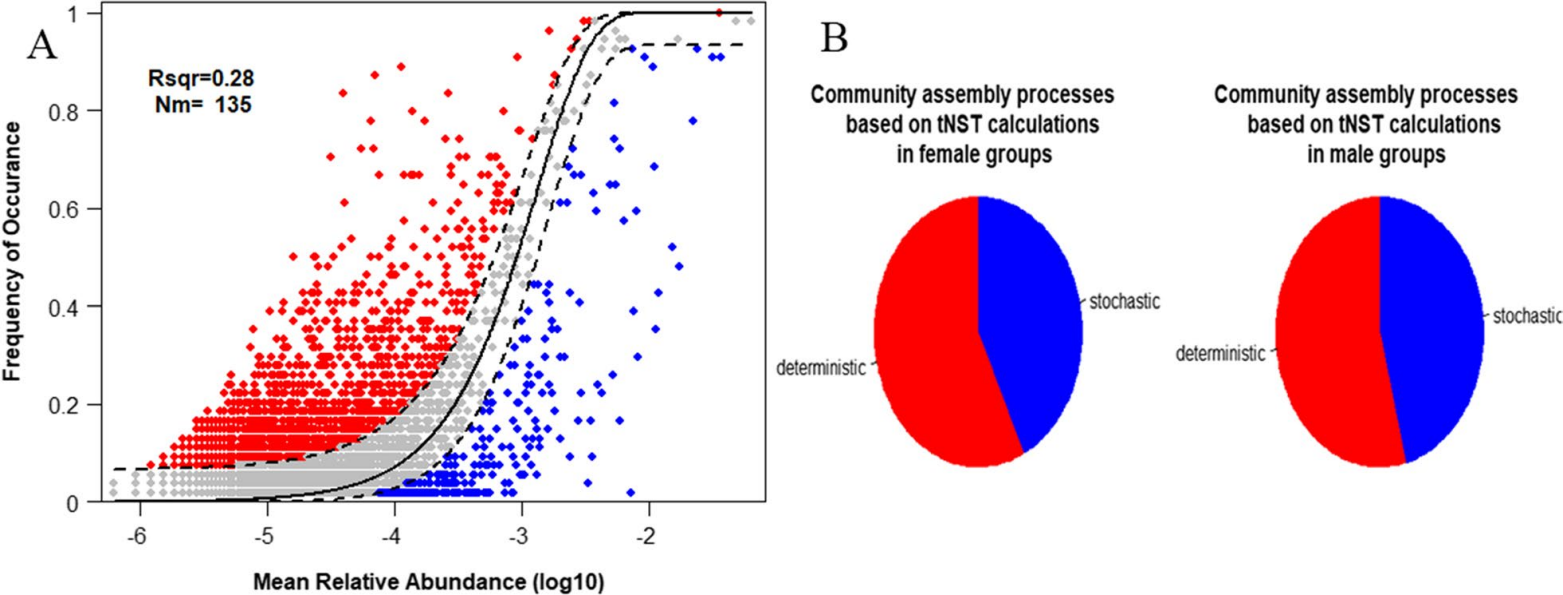
The analysis of community assembly mechanism. **A** The neutral community model of community assembly. The solid black lines represented the most fit to the neutral model, and the dashed lines represent 95% confidence intervals around the model. Red, grey and bule plots represented the occurrence frequency of OTUs above prediction, fit prediction and below prediction, respectively. R^2^ remarked the fitness of neutral community model. **B** The results of the normalized stochasticity ratio (NST) in female and male group, with no significant differences (p > 0.05)

The gut microbial community assembly mechanism was analyzed. The normalized stochasticity ratio (NST) was performed to assess the role of stochastic and deterministic processes in bacterial community assembly (Fig. 4B). It was observed that the distribution of NST values was below the 50% threshold line for both the female and male groups of microbial communities, indicating the dominance of deterministic processes in both groups of communities. Moreover, there was no significant difference in the NST values between the female (an average of 42.77%) and male (an average of 45.87%) groups (Wilcox Test p > 0.05). Additionally, the neutral community model (NCM) was used to show the relative importance of the neutral process (Fig. 4A). The R^2^ represents the overall goodness of fit of the neutral community model. A low R^2^ (0.28) was overserved in this study that indicating it is not close to the neutral model, and the community construction was more likely influenced by the deterministic process and rarely influenced by the stochastic process.

### The BMI-related gut microbiome

A relative abundance analysis for each bacterial phylum and bacterial genus in different BMI groups was performed. Considering the sex-related gut microbial differences, male and female individuals were analyzed separately, and the subjects were divided into six groups: L-F, N-F, H-F, L-M, N-M, and H-M. The various α diversity indices were calculated to show the gut bacterial diversity in different groups. However, no significant difference in α diversity was observed in each BMI group (p > 0.05). The relationship between BMI and gut bacterial diversity was investigated with Pearson correlation. A slightly negative correlation was observed between BMI and gut bacterial α diversity, though not reaching significant difference in each group [Observed_species (R = − 0.12, p = 0.39), Shannon index (R =  − 0.17, p = 0.23) and Simpson index (R = − 0.15, p = 0.28)] (Additional file 1: Fig. S3). The difference in gut bacterial taxon was analyzed at bacterial phylum and genus level in each BMI group. The results of male and female subjects were shown separately.

**Fig. 5:**
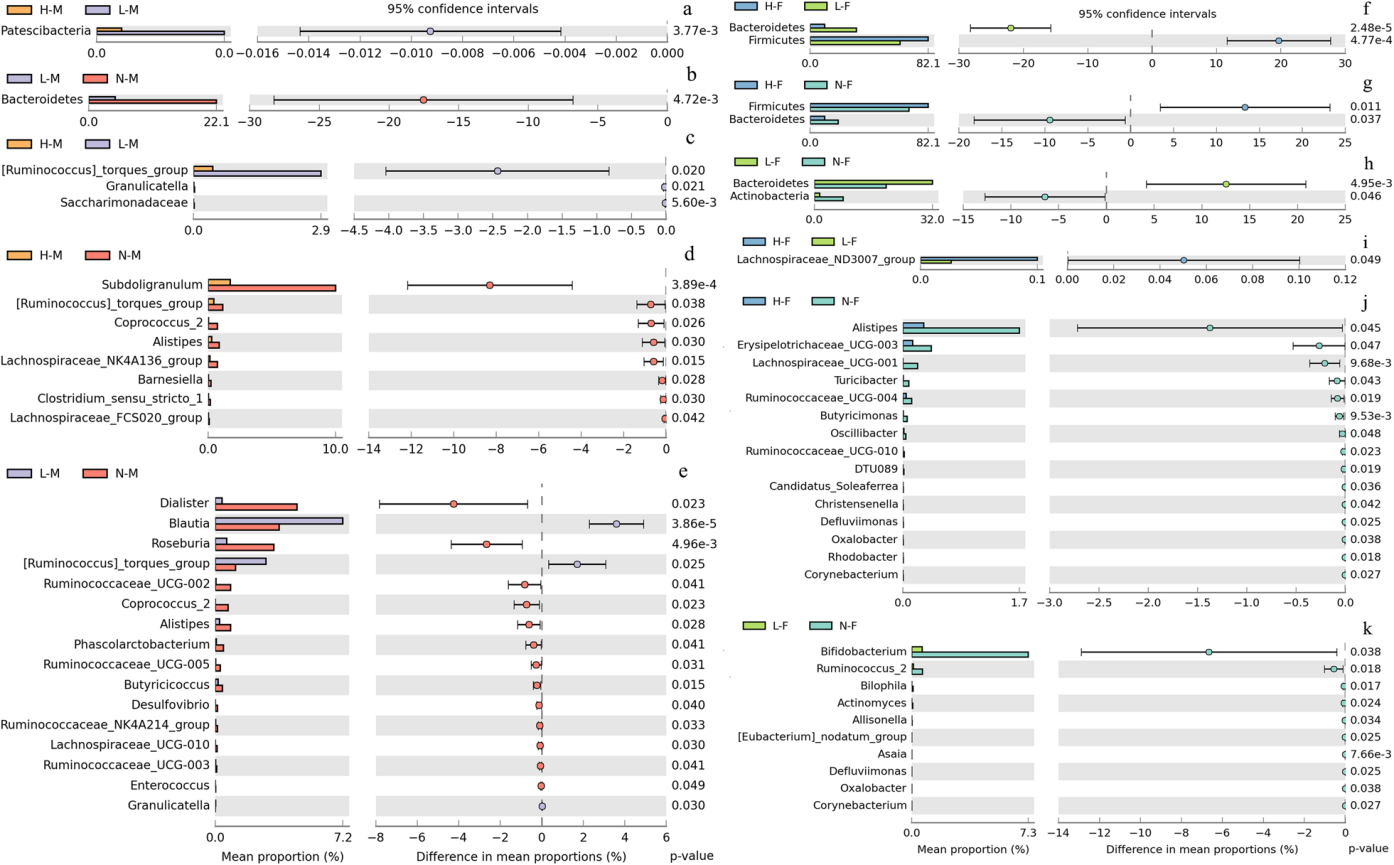
Gut microbiota comparison in different BMI groups at phylum and genus level. The significant phyla and genre with significant richness difference (*p* < 0.05, computed by STAMP) in each group were shown. **a**, **b**, **f**–**h** were the significant difference at phylum level. **c**–**e**, **i**–**k** were the significant difference at genus level. H-M: overweight male group; N-M: normal weight male group; L-M: underweight male group; H-F: overweight female group; N-F: normal weight female group; L-F: underweight female group

In male subjects, the taxonomic difference in the gut bacterial community at phylum and genus level was manifested in Fig. 5 which showed there were remarkable differences in each BMI group. In detail, at the phylum level, increased representative phyla were *Patescibacteria* (p < 0.05) in the underweight group and *Bacteroidetes* (p < 0.05) in the normal-weight group (Fig. 5a, b). At the genus level, comparing the microbial changes between overweight and underweight groups, it was found that the abundance of 3 genera in the underweight group increased significantly (Fig. 5c), and the distinction between the overweight and normal-weight group showed a great increase in the abundance of 8 genera in normal-weight subjects (Fig. 5d), while the collation of underweight and normal-weight subjects revealed a remarkable increase in the abundance of 13 genera in normal-weight subjects and 3 genera in underweight subjects (Fig. 5e).

Within the female group, the taxonomic distinction in the gut bacterial community at phylum and genus level was also revealed notable differences across BMI groups. Specifically, at the phylum level, the significantly different phyla were *Bacteroidetes*, *Firmicutes,* and *Actinobacteria* (Fig. 5f–h). At the genus level, comparing the microbial changes between overweight and underweight groups, the abundance of 1 genus in the overweight group increased significantly (Fig. 5i), and the collation of the overweight and the normal-weight group showed a remarkable increase in the abundance of 15 genera in normal-weight subjects (Fig. 5j), while the distinction between underweight and normal-weight subjects revealed a great increase in the abundance of 10 genera in normal-weight subjects (Fig. 5k).

### Discrimination of different BMI groups

**Fig. 6:**
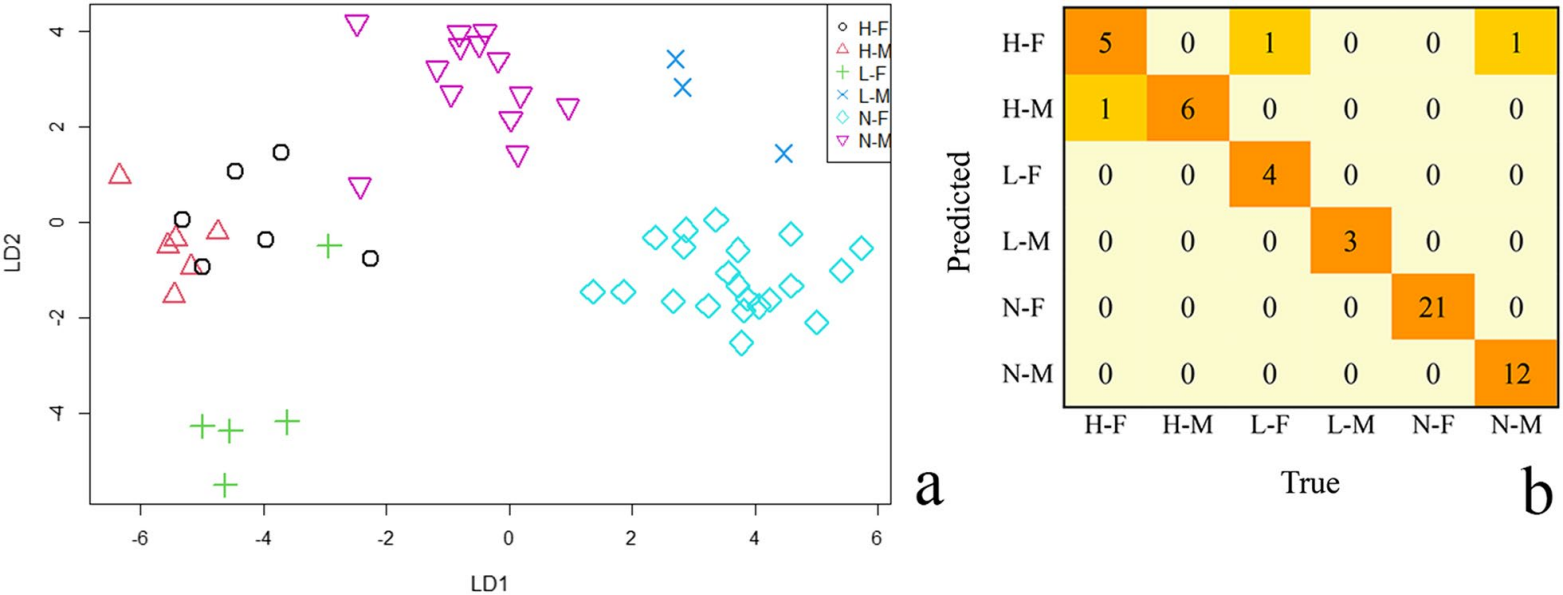
Discriminate the subjects of six different BMI groups by Fisher’s Linear Discriminant analysis (LDA). **a** A clear separation among six BMI groups in the liner discriminant plot. **b** The evaluation of model’s goodness-of-fit that had a correct rate of 94.4% and only three subjects were not correctly identified

According to the comparison of gut bacterial community composition in each BMI group at the genus level, 44 genera were found to be unique (Fig. 5). A Fisher’s Linear Discriminant Analysis (LDA) based on the 44 specific genera was performed to define a model to discriminate the subjects in six different BMI groups. Six bacteria were excluded due to the consistency within groups, and 38 bacteria were left for LDA analysis finally. The goodness-of-fit of the discriminant model was measured using the resubstitutaion method. By Fisher’s Linear Discriminant analysis, ninety- four point four percent (94.4%) of original BMI grouped subjects were correctly classified. According to the model’s goodness-of-fit, we found 3 subjects were incorrectly predicted (Fig. 6b). The linear discriminant plot was shown in Fig. 6a, which revealed a clear separation across six BMI groups. In detail, it illustrated an obvious classification among L-M, N-M, L-F, and N-F groups, with the majority of samples within each group clustered together with a clear distinction. However, there were two overlapping points in the H-F and H-M groups. One sample in the L-F group and one in the N-M group overlapped with the H-F group. These overlaps also explained why the model incorrectly predicted three samples.

### The prediction of body type

**Fig. 7:**
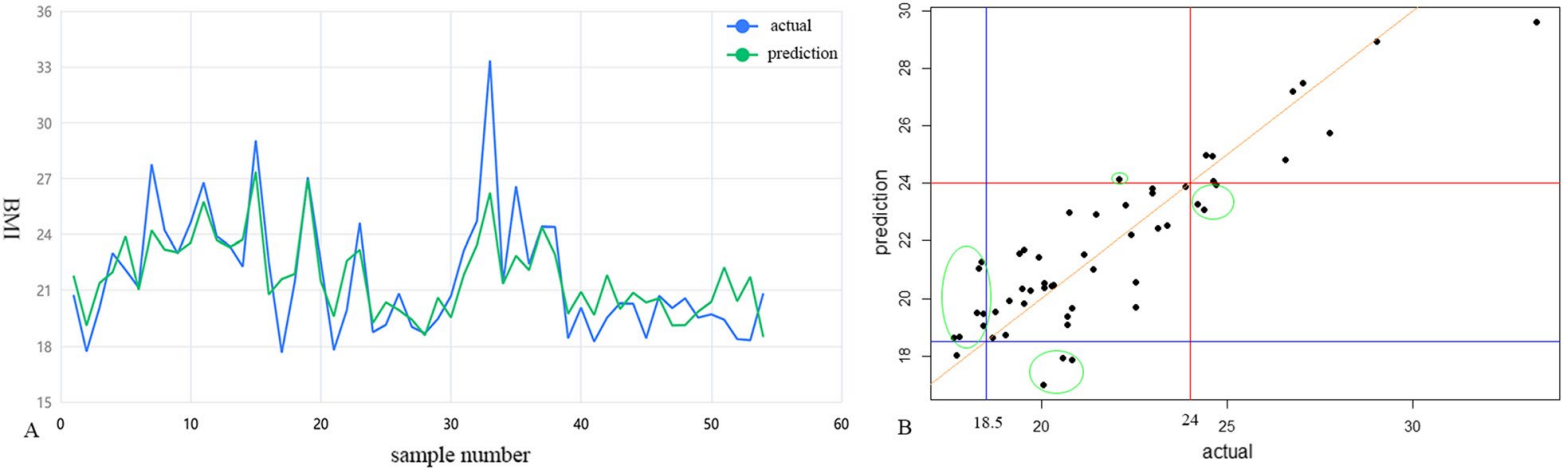
The prediction of body type by regression analysis. **A** The results of the ridge regression model. Body type was predicted correctly for 72% of samples. **B** The results of linear regression model. The blue line was BMI = 18.5 kg/m^2^; the red line was BMI = 24 kg/m^2^; the orange line represented y = x, and the smaller the vertical distance from the point to the line, the closer the predicted value is to the actual value; the dots in green elliptical represented the wrong prediction of body type (14 samples). Seventy four percent (74%) of samples were predicted correctly

Regression analysis was performed to evaluated the relationship between BMI and gut bacteria. The regression analysis was carried out using 44 specific genera (see above). Ridge regression analysis was first conducted. The prediction was shown in Fig. 7A. The *R*^2^ of the model was 0.663 and the model performed relatively well. Seventy two percent (72%) of samples were predicted correctly. Then the linear regression model was performed after reducing noise in dataset. The actual BMI value and prediction were also shown in Fig. 7B. The Multiple *R*^2^ of the linear regression model was 0.7837 and the adjusted *R*^2^ was 0.5223 (*p* = 0.0038), with the range of residuals was between − 2.8858 and 3.7375. Body type was predicted correctly for seventy four percent (74%) of samples by the linear regression model.

## Discussion

Microbial typing of fecal samples can play a vital role in forensic practice, but the research on its application in forensic personal identification is still lacking. In this study, the gut microbiota composition of unrelated subjects was examined to explore whether individual characteristics of the gut microbiota could be applied to forensic practice. Various factors that affect the gut microbiota. Sex maturation and sex hormones perform critical roles after puberty, and the estrogen, testosterone, and androgen are proven to be related to the gut microbiome have been reported [29]. Moreover, increased reports have revealed the relationship between gut microbiota and obesity [30–32]. The indicator of obesity is body mass index, which is closely related to body type. It seems sex and body mass index could be suitable traits to apply gut microbiota to forensic investigation. So, the subjects were grouped according to the two characteristics in the gut microbiota composition analysis. Two variables, age and region, were controlled when collecting the samples.

In the analysis of gut microbiota and sex, significant differences had been revealed between female and male gut microbiota in α diversity and bacterial community composition. From the results of LEfSe, *Proteobacteria* and *Synergistetes* were the biomarkers with significant differences in males and females, respectively. At the genus level, there were five biomarker genera (*Anaerofusis, Intestinibacter,* etc*.*) of gut microbiota in females and three in males (*Escherichia_shigella*, *Rodentibacter, Caproiciproducens*). These results indicated the gut microbiota composition had a sex-related variation. Previous studies have also shown a differential gut microbial community composition between males and females, but the unique bacterial genera are diverse. The previous study based on the human microbiome project had shown community type D was characterized in males gut microbiota with *Prevotella* in a high relative abundance, while community type C microbial genera contained *Ruminococcaceae*, *Alistipes*, *Faecalibacterium* were reparented in females [12]. Besides, in the gut microbial studies of Dutch, Spanish, and Japanese populations, the results as well as showed a differential gut microbial composition between males and females, but the unique bacterial genera of the differences were not completely consistent with each other that were shown in Table 1 [18, 33, 34]. A higher α diversity in the female gut microbiota than in the male was revealed in this study (p > 0.05), which was consistent with a study of 1135 subjects in which females showed greater gut microbial diversity than males[34], while other studies showed no statistically significant difference[18, 33] (Table 1). These inconsistent phenomena may be caused by the complexity of factors included region and age that affect the gut microbiota. A series of changes of gut community composition have been revealed associated with age [18]. The variable of age was controlled in our study, while the subjects were distributed between 20 and 89 years of age in other studies. In addition, there is a study has shown differences in the gut microbiota in diverse US populations that may be caused by dietary acculturation [35]. These could also explain the inconsistent results of sex-related gut microbial genera and sex differences in gut microbial diversity in various studies. On the one hand, sex differences in gut microbiota may be hormonally related. Estrogen and testosterone, as a manifestation of sex discrimination, have been shown to directly affect the gut microbiota [29, 36]. This sex-related gut microbial difference is reflected not only in the age group with high sex hormone secretion but also in all age groups [33, 37]. On the other hand, the various immune system and functions in different sex subjects might affect the human gut microbial community composition [38]. The human immune system and the physiological activities of hormones are all regulated by genes. It’s wise to regard the gut microbiome as shaped by human genetics [16]. Thus, this difference in gut composition caused by human genes facilitates the application of gut microbiota to individual portraits in forensic practice. Two methods (NST and NCM) were used to evaluate the gut bacterial community assembly mechanism. The results of the two approaches were both suggested that deterministic processes played a vital role in shaping the gut bacterial community assembly. The influence of deterministic processes on the two sex groups of communities was almost equal, which may explain why male and female subjects cannot be completely distinguished based on BC distance (Fig. 1). Therefore, the other personal trait has been included in the research.

**Table 1:** The comparison of sex-related gut microbiota composition differences in various country

Country	Males	Females	α diversity	Age of subjects	References
China	*Escherichia_shigella*,*Rodentibacter, Caproiciproducens*	*Anaerofusis, Intestinibacter*	F > M	20–30	This study
Japan	*Prevotella, Megamonas, Fusobacterium, Megasphaera*	*Bifidobacterium, Ruminococcus, Akkermansia*	NS^*^	20–89	[18]
Netherlands	12 specific microbial species were significantly associated with sex, including *Akkermansia muciniphila, Lachnospiraceae bacterium, Subdoligranulum, Eggerthella, Collinsella aerofaciens, Eubacterium eligens, Gordonibacter pamelaeae, Lachnospiraceae bacterium, Bifidobacterium longum, Alistipes, onderdonkii, Bilophila*		F > M	18–81	[31]
Spanish	*Clostridium, Coprococcus, Dorea, Lachnospira, Roseburia, Veillonella*	*Bacteroides, Barneciellaceae, Butyricimonas, Parabacteroides, Rikenellaceae*	NS	20–75	[30]

NS represented no statistically significant differences

The body types, as one of the important individual features, could help police narrow down suspects in the practical application of forensic science. BMI was identified as a suitable proxy for determining the percentage of body fat in a population by Ancel Keys in 1972 [39], and subsequently became a criterion for differentiating weight levels, closely related to body types. As an appearance data depicting offenders, BMI is also associated with the composition of the gut microbiota [19, 31]. Given that the sex differences in gut microbiota observed above, male and female subjects were compared separately in the study of BMI and gut microbiota. No significant differences were revealed in the comparison of α diversity. A negative correlation between α diversity and BMI was shown in a study exploring the gut microbiota and BMI in Chinese male college students [30], and a weak negative correlation was also shown between α diversity and BMI in our study, although it did not reach a significant difference. The sample distribution was uneven and the subjects were mainly concentrated in the normal-weight group in our study, which may have contributed to a p-value of the Pearson test greater than 0.05. In a comparison of bacterial composition at the phylum level, significant differences in the relative abundance of *Firmicute* in both male and female overweight groups have existed, and the underweight and normal-weight group of *Bacteroidetes* showed remarkably different relative abundances within the female group. However, unfortunately, a non-meaningful positive correlation had not been shown (p > 0.05, not shown) in the correlation analysis between F/B ratio and BMI, and it was also shown to be uncorrelated with BMI in the study by Tomohisa Takagi et al. [18]. It was conflicted with the correlation between the F/B ratio and BMI in other studies [30, 40], which may be due to the external factors contained habitat, dietary acculturation, psychical exercise, social pressure, etc.

To apply the gut microbiota to forensic practice, forensic scientists are keen on whether different individuals/populations can be distinguished or whether some individual signature can be predicted by the characteristics of gut microbiota to provide clues for investigation and evidence for trial. Ninety-four point four percent (94.4%) of the subjects in six BMI groups were correctly classified by LDA analysis based on 38 unique genera, which suggested that it was possible to distinguish various individuals using bacterial genera with high differences. In addition, there was an interesting observation. According to the linear discriminant plot, we found that male and female subjects in the normal weight and underweight group were clustered together separately showing a clear difference, which indicated that it was wise to separate male and female subjects for analysis. However, there were some overlaps in the overweight group. This may indicate that sexual differences were not obvious in the overweight group, and the gut microbial differences were dominated by some bacterial genera associated with obesity. It seemed the LDA analysis model based on bacterial genera with high differences had a good fit and could classify most individuals correctly. In addition to classification, we also attempted regression analysis. From the analysis results of BMI-related gut microbiome, the correlation between BMI and gut bacterial diversity had not reach a significant difference. So, the regression analysis was carried out using 44 specific genera. Though the *R*^2^ of the ridge regression model showed it performed relatively well, the *p* value of the ridge regression was greater than 0.05 and adjusted *R*^2^ was low. It may be due to the inclusion of some features in the model that are not statistically relevant. Thus, the lasso regression was first performed to keep the model concise and reduce noise in the dataset from variables without statistically relevant, and then linear regression model was constructed. The final linear regression analysis model showed an accuracy of seven four percent (74%) in predicting body type. Whereas, the small number of subjects in our study, especially in the overweight and underweight group, prevented us from dividing the data into validation and test sets to perform predication, which made the predictive effect of the model was not validated. Both of the results of classification and regression analysis suggested that the different body types can be distinguished by analyzing gut microbiome, and that the specific bacteria of the gut microbes can be used to predict the body type (BMI) of an individual. It seems possible to portray human features by the gut microbiome, which is exactly what forensic scientist are interested in that may be can used in forensic application. In some special cases of low human DNA amounts or degraded DNA or twins, involving fecal trace, it is hard to get full DNA information from feces, however, the giant microbial information in feces can provide useful clues for criminal investigations. The prediction of body type is one of clues to narrow down the suspects. Two factors (sex and BMI) were explored in this study, the analysis results of human portraits are more comprehensive and reasonable if more influencing factors are taken into consideration, which needs to be achieved based on a large number of population data of gut microbial sequencing, and combined with machine learning.

Gut microbiome may have great potential for using in forensic investigation. However, diverse environmental factors (temperature, moisture, pH, micro bacteria, etc.,) can influence the biological specimens. These environmental factors usually are controlled in research as much as possible, but it’s highly unpredicted in actual forensic cases. In addition, some articles postulating a potential association between gut microbiota and obesity have conflicting results and have not been duplicated in clinical studies [41–44], and the specific phyla and genera determined are inconsistent in different study. The reason may be the multifactor that can impact the gut microbiome, and the differences in the processing of multiple steps comprising extraction, amplification and sequencing, which can cause the inconsistent of bacterial taxa abundance. These divergent findings block the identification of consistent patterns of human gut microbiota associated with personal signature. In total, it is still challenging to applying microbial taxa for forensic personal identification. The individual differentiation using gut microbiota needs further study.

## Conclusion

In summary, a series of individual factors included sex, BMI, etc. affect the composition of gut microbiota. These factors make each individual have a unique gut microbiota. In our study, LDA analysis based on distinctive bacterial genera was able to correctly classify 94.4% of subjects into different BMI groups, and the linear regression analysis model showed an accuracy of seven four percent (74%) in predicting body type, which seems that it is possible to use the gut microbiota to make inferences about individual characteristics.

## Supplementary Information

**Additional file 1: Figure S1.** The α diversity of gut microbiota in male and female subjects. The sex group did differ significantly in Chao1 index, the number of observed species (richness estimation) and Shannon index (evenness estimation) (wlicox test, *p* = 0.029, *p* = 0.028 and *p* = 0.036, respectively). **Figure S2.** The relative high abundance bacteria of male and female subjects at phylum and genus level. At phylum level, the predominant phyla in gut bacterial community were *Firmicutes, Bacteroidetes, Actinobacteria, Proteobacteria.* At genus level, the relative high abundance genera were *Faecalibacterium, Bacteroides, Subdoligranulum, Escherichia-Shigella, Blautia, Prevotella_9, Agathobacter, Bifidobacterium, Roseburia, Dialister.* **Figure S3.** The correlation between BMI with gut microbial α diversity: Observed_species(a), Shannon index (b) and Simpson index (c) derived by Pearson correlation. A slightly negative correlation was observed between BMI and gut bacterial α diversity, though not reaching significant difference in each group (Observed_species (R = -0.12, p = 0.39), Shannon index (R = -0.17, p = 0.23) and Simpson index (R = -0.15, p = 0.28).

## Data Availability

The dataset supporting the conclusions of this article had been deposited in the NCBI Sequence Read Archive repository under the accession number PRJNA777123 (https://www.ncbi.nlm.nih.gov/bioproject/PRJNA777123).
