# Supplementary material for: Assess the diversity of gut microbiota among healthy adults for forensic application
Source: Microb Cell Fact. 2022 Mar 24;21:46. doi: 10.1186/s12934-022-01769-6 (PMC8944125; doi:10.1186/s12934-022-01769-6)

Figure S1. The α diversity of gut microbiota in male and female subjects. The gender group did differ significantly in Chao1 index, the number of observed species (richness estimation) and Shannon index (evenness estimation) (wlicox test, *p*=0.029, *p*=0.028 and *p*=0.036, respectively).


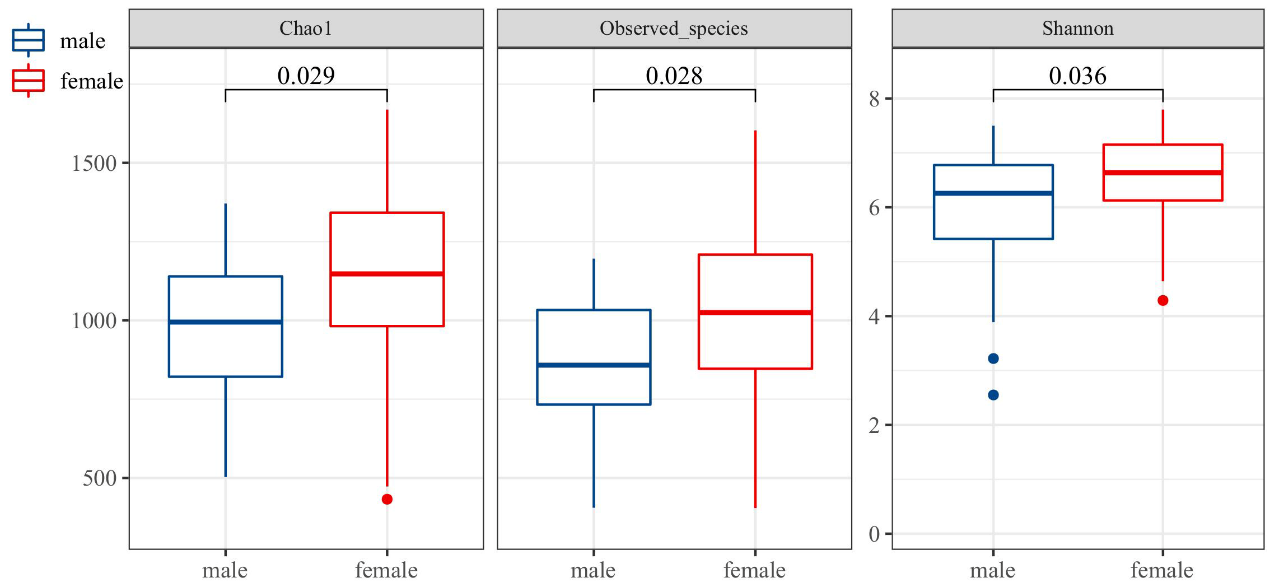


Figure S2. The relative high abundance bacteria of male and female subjects at phylum and genus level. At phylum level, the predominant phyla in gut bacterial community were *Firmicutes, Bacteroidetes, Actinobacteria, Proteobacteria.* At genus level, the relative high abundance genera were *Faecalibacterium, Bacteroides, Subdoligranulum, Escherichia-Shigella, Blautia, Prevotella_9, Agathobacter, Bifidobacterium, Roseburia, Dialister.*


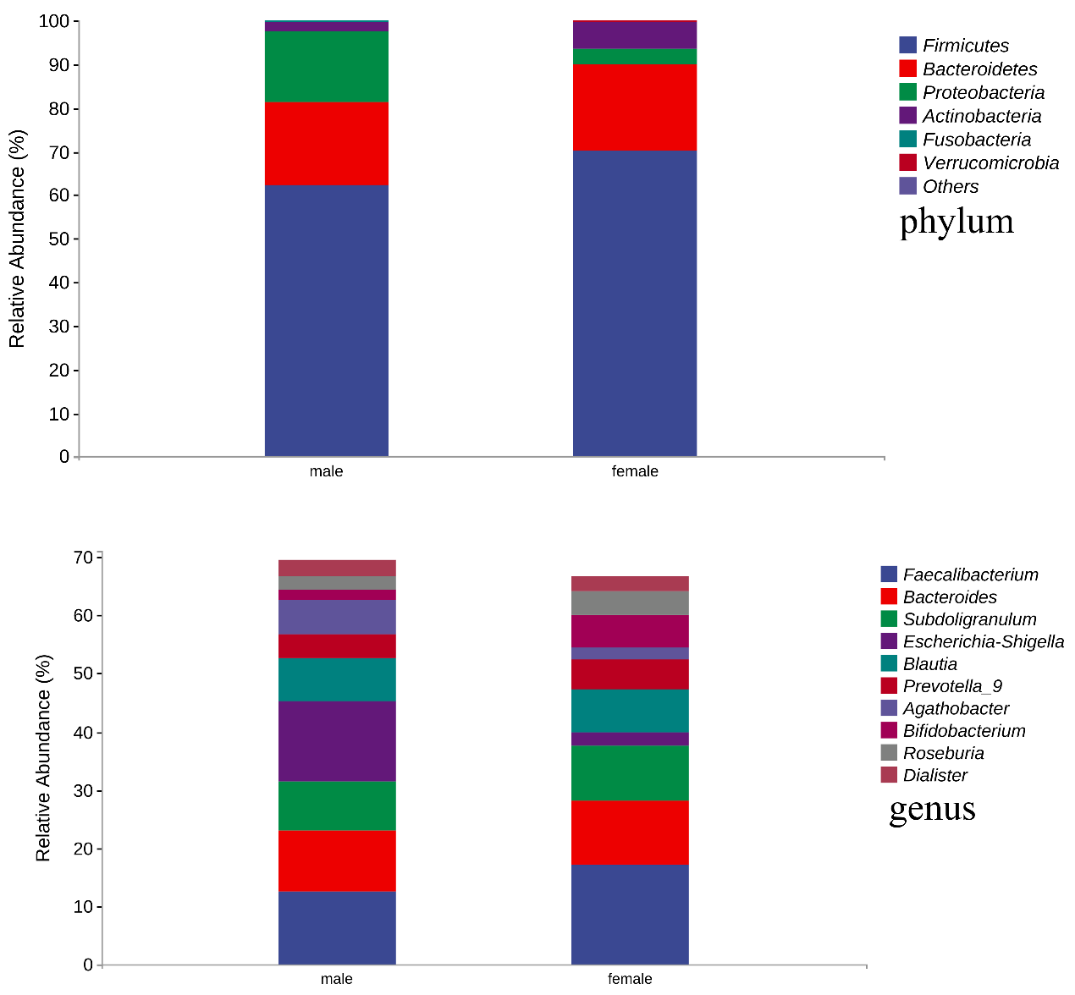


Figure S3. The correlation between BMI with gut microbial α diversity: Observed_species(a), Shannon index (b) and Simpson index (c) derived by Pearson correlation. A slightly negative correlation was observed between BMI and gut bacterial α diversity, though not reaching significant difference in each group (Observed_species (R=-0.12, p=0.39), Shannon index (R=-0.17, p=0.23) and Simpson index (R=-0.15, p=0.28).


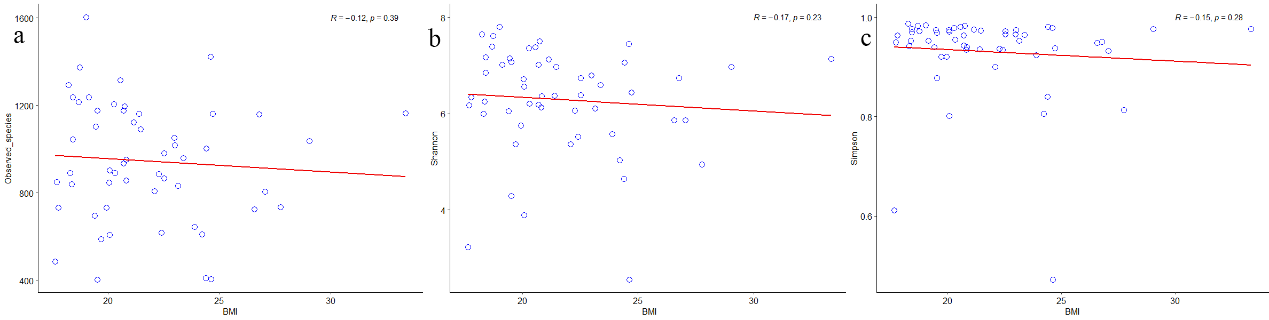

Supplement: Supplementary file 1 — Additional file 1: Figure S1. The α diversity of gut microbiota in male and female subjects. The sex group did differ significantly in Chao1 index, the number of observed species (richness estimation) and Shannon index (evenness estimation) (wlicox test, p = 0.029, p = 0.028 and p = 0.036, respectively). Figure S2. The relative high abundance bacteria of male and female subjects at phylum and genus level. At phylum level, the predominant phyla in gut bacterial community were Firmicutes, Bacteroidetes, Actinobacteria, Proteobacteria. At genus level, the relative high abundance genera were Faecalibacterium, Bacteroides, Subdoligranulum, Escherichia-Shigella, Blautia, Prevotella_9, Agathobacter, Bifidobacterium, Roseburia, Dialister. Figure S3. The correlation between BMI with gut microbial α diversity: Observed_species(a), Shannon index (b) and Simpson index (c) derived by Pearson correlation. A slightly negative correlation was observed between BMI and gut bacterial α diversity, though not reaching significant difference in each group (Observed_species (R = -0.12, p = 0.39), Shannon index (R = -0.17, p = 0.23) and Simpson index (R = -0.15, p = 0.28). [file 12934_2022_1769_MOESM1_ESM.docx]
